# Functional similarities of microRNAs across different types of tissue stem cells in aging

**DOI:** 10.1186/s41232-018-0066-9

**Published:** 2018-06-06

**Authors:** Koichiro Watanabe, Yasuaki Ikuno, Yumi Kakeya, Hirotaka Kito, Aoi Matsubara, Mizuki Kaneda, Yu Katsuyama, Hayato Naka-Kaneda

**Affiliations:** Department of Anatomy, Shiga University of Medical Science, Seta Tsukinowa-cho, Otsu, Shiga 520-2192 Japan

**Keywords:** Stem cell aging, microRNA, miR-17, miR-125b, miR-181a, SASP

## Abstract

Restoration of tissue homeostasis by controlling stem cell aging is a promising therapeutic approach for geriatric disorders. The molecular mechanisms underlying age-related dysfunctions of specific types of adult tissue stem cells (TSCs) have been studied, and various microRNAs were recently reported to be involved. However, the central roles of microRNAs in stem cell aging remain unclear. Interest in this area was sparked by murine heterochronic parabiosis experiments, which demonstrated that systemic factors can restore the functions of TSCs. Age-related changes in secretion profiles, termed the senescence-associated secretory phenotype, have attracted attention, and several pro- and anti-aging factors have been identified. On the other hand, many microRNAs are linked with the age-dependent dysregulations of various physiological processes, including “stem cell aging.” This review summarizes microRNAs that appear to play common roles in stem cell aging.

## Background

Overcoming age-related diseases and elongating the healthy lifespan are emerging issues for aging societies. Dysfunctions of aged tissue stem cells (TSCs) contribute to loss of tissue homeostasis, including reductions in lymphopoiesis and the long-term repopulating abilities of hematopoietic stem/progenitor cells (HSCs) [1, 2], the muscle repair capacity of skeletal muscle satellite cells [3], and the multipotency of mesenchymal stem/stromal cells (MSCs) [4]. The restoration of TSC functions in murine heterochronic parabiosis experiments triggered interest in the rejuvenation of aged TSCs [5]. Thereafter, several pro-aging [6–12] and anti-aging [13–16] systemic factors were identified, although some of the findings are conflicting [17]. Senescent cells secrete a myriad of inflammatory factors, referred to as the senescence-associated secretory phenotype (SASP) [18]. Clearance of senescent cells delays the induction of various geriatric pathologies, supporting the concept that the SASP promotes aging in a non-cell-autonomous fashion [19, 20]. Several lines of evidence indicated that age-related TSC dysfunctions and tissue-level pathologies can be improved by manipulating (reversing) cell-extrinsic/systemic conditions, at least in part.

We previously identified growth differentiation factor 6 (Gdf6; also known as Bmp13 and CDMP-2) as a regenerative factor secreted by young MSCs [21, 22]. Upregulation of human GDF6 restores the differentiation potential of old MSCs in vitro and reverses multiple age-related pathologies in vivo. miR-17 and its paralogues miR-106a and 106b (miR-17/106) regulate not only differentiation potential but also expression of some secretory factors, including Gdf6, and are implicated in the decline of these functions with age. Many microRNAs are associated with age-related dysfunctions of several types of TSCs. Here, we review microRNAs, which are commonly downregulated with age and induced dysregulation of cytogenesis, proliferation, and inflammation in multiple TSCs, and discuss functional similarities of microRNAs across different types of TSCs in aging.

### miR-17 family (miR-17-92a, 106b-25, and 106a-363 clusters)

miR-17 family members play essential and pleiotropic roles in development, metabolism, diseases, tumorigenesis, and aging [23, 24]. We first identified miR-17/106 family members as key regulators of the neurogenic-to-gliogenic switch in developing neural stem/progenitor cells (NSCs) by controlling the “competence” necessary for NSCs to respond to gliogenic cell-extrinsic signals [25, 26]. Next, we found that downregulation of miR-17/106 induces a decline in differentiation potential and dysregulated expression of secretory factors in old MSCs [22]. Another group also reported a relationship between miR-17/106 and an age-dependent decrease in the osteogenic potential of MSCs [27]. miR-17/106 also regulate the proliferation and development of HSCs [28–30]. Other reports studied the impact of miR-17 overexpression in vivo. Transgenic mice expressing miR-17 exhibit delayed tissue growth and have an elongated lifespan [31, 32]. Epidemiologic studies reported that miR-17 family members are upregulated in centenarians, which supports the hypothesis that these microRNAs are important for the young healthy conditions and involved in human aging [33, 34].

### miR-125b

A myeloid skewing phenotype and a decline in engraftment capability have long been recognized as age-related dysfunctions of old HSCs [1]. miR-125b is expressed in HSCs, and overexpression of miR-125b predominantly expands lymphoid-biased HSCs [35]. In addition, miR-125b can increase the level of myeloid progenitors [36]. Both reports showed that miR-125b overexpression increases the engraftment capabilities of HSCs and progenitors in transplantation assays into irradiated mice. Moreover, reduction of miR-125b increases expression levels of the chemokine CCL4 with age [37]. miR-125b activates and is activated by the NF-κB pathway [38, 39] is sometimes regarded as an “inflamma-miR,” which is implicated in the regulation of immune and inflammatory responses [40]. miR-125b directly suppresses p53 expression in developing NSCs. miR-125b is expressed throughout zebrafish embryos and is enriched in the brain, while loss of miR-125b elevates p53 expression and triggers p53-dependent apoptosis in these embryos [41]. miR-125b is also expressed in MSCs [42], epidermal stem cells [43], and some types of tumor cells [44–48]. Interestingly, *lin-4*, a *Caenorhabditis elegans* homolog of miR-125b, is a heterochronic gene and generates the temporal pattern of many cell lineages during development [49], and is related to lifespan and tissue aging via its control of the insulin/insulin-like growth factor–1 pathway [50]. Overexpression of *lin-4* elongates lifespan, whereas loss-of mutation accelerates tissue aging and shortens it.

### miR-181 family (miR-181a/b/c/d)

Chronic inflammation accelerates systemic aging [10]. miR-181 family members have anti-inflammatory functions and are categorized as inflamma-miRs, together with miR-125b [40]. miR-181 regulates the differentiation of multiple types of TSCs, such as HSCs [51], myoblasts (activated progenitor cells) [52], MSCs [53], and some types of cancer stem cells [54–56]. We confirmed that miR-181 family members are downregulated with age in multiple TSCs (HSCs, MSCs, and intestinal stem cells). However, they continue to be expressed in differentiated cells and function pleiotropically. The age-dependent decline in miR-181a expression induces functional defects in CD4+ T cells [57]. miR-181a is downregulated in old pancreatic beta cells and necessary for their proliferation [58]. Extracellular vesicles derived from brain metastatic cancer cells contain miR-181c and can destroy and pass through the blood-brain barrier [59]. The critical roles of miR-181 in age-related cell-intrinsic dysfunctions of TSCs are unclear. The old TSCs with downregulated miR-181 family members would generate abnormal somatic cells, which have something dysfunctions, and these cells may contribute to the disturbance of tissue homeostasis.

### Commonality of microRNA functions among various types of TSCs

**Fig. 1:**
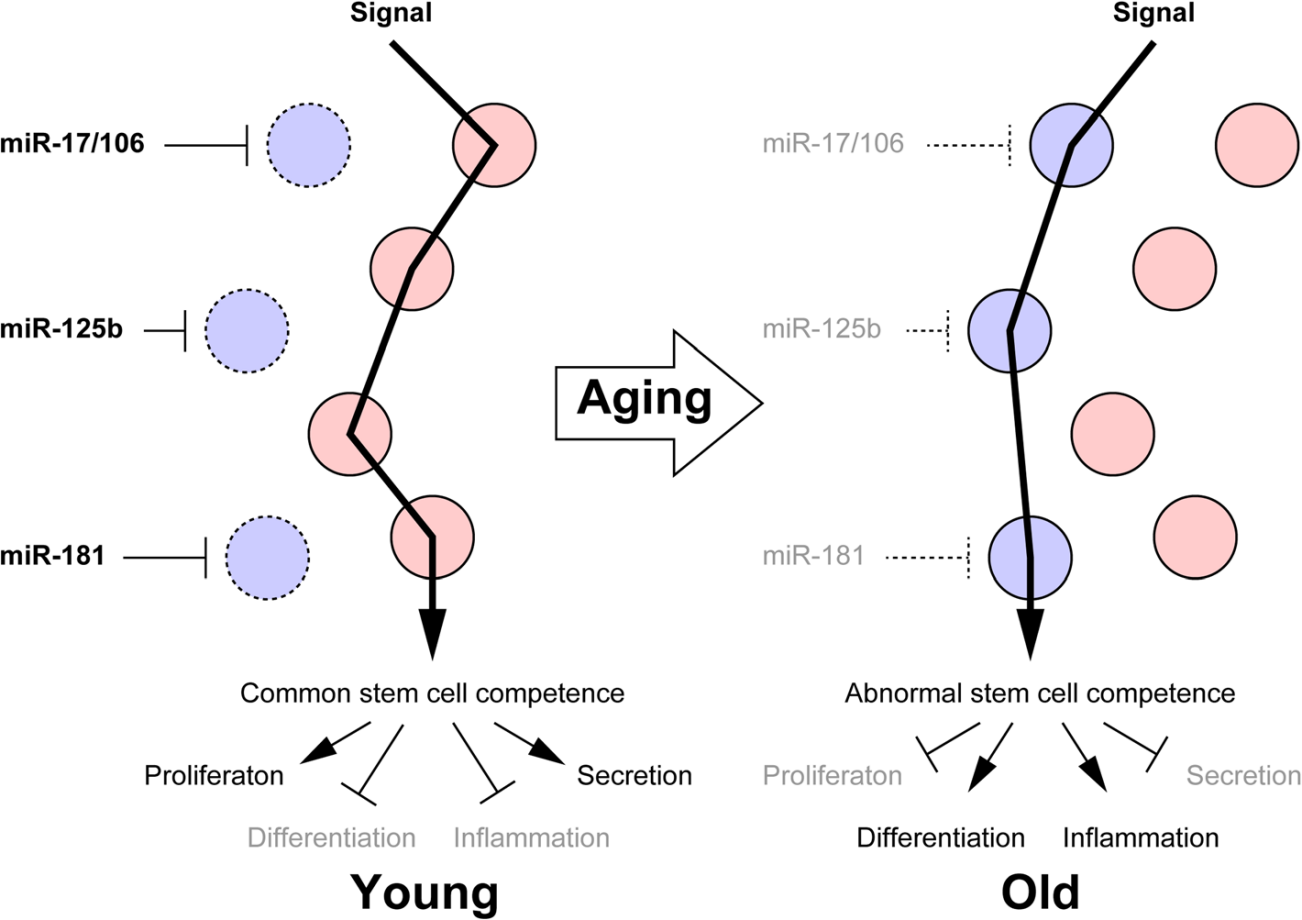
Schematic diagram of the disruption of microRNA-mediated stem cell competence. Decline in microRNAs for regulation of stem cell functions induces disruption of proper stem cell competence and dysfunctions

Recent studies have revealed that a part of microRNAs appear to play common roles in stem cell aging (Table 1). In fact, many microRNAs, including miR-17 family, miR-125b, and miR-181 family members, show similar expression pattern, namely they are expressed at higher levels during proliferating phase and downregulated with age. This is supported by a report concerning the classification of tumor cells derived from various tissues based on their microRNA, not their mRNA, expression profiles, suggesting that the existence of functionally common microRNAs, at least, for proliferation and undifferentiated states [60]. We have focused on microRNA-mediated “competence regulation,” which is responsible for the responsiveness to the various cell-extrinsic signals, as the fundamental machinery controlling the properties of TSCs, and miR-17 family members are key regulators in this context [22, 25, 61]. In our previous study, we revealed that miR-17/106 switches the usages of JAK-STAT and BMP pathways from neurogenic to gliogenic signals [25]. In young states, microRNAs regulate signal transduction correctly. Downregulation of microRNAs with age should induce deregulation of signal transduction and reflect abnormal phenotypes to signals (Fig. 1). All miR-17, miR-125b, miR-181 family members are downregulated various old TSCs and downregulation of them suppresses cytogenesis, proliferation, and secretion of homeostatic factors and promotes inflammation and tumorigenesis (Table 1).

**Table 1:** Functional similarities of microRNAs in different types of TSCs

microRNA family	Differentiation (specification)	Proliferation, survival, and apoptosis	Secretion and inflammation	Tumorigenesis	Others
miR-17/106	MSCs (↑Ad, ↑Os) [22, 27] NSCs (↑N, ↓G) [25] HSCs (↑B, ↑Ly, ↑My) [28–30]	↑HSCs [28–30]	MSCs (↑Gdf6 and etc.) [22]	Lymphoma [28–30]	
miR-125b	HSCs (↑Ly, ↑My) [35, 36]MSCs (↑Ad, ↑Os) [42] Skin stem cells (↓Epi, ↓Oil, ↓HF) [43]	↑HSCs [35, 36]↑NSCs [41] ↑Skin stem cells [43]	HSCs (↓CCL4, ↑NF-κB, ↓TNFAIP3) [37, 39, 40]	Breast cancer [44] Hepatocellular carcinoma [45] Leukemia [46] Skin tumor [47] Stomach adenocarcinoma [48]	↑HSC engraftment [35, 36]
miR-181	HSCs (↑Ly) [51] Myoblasts (↑Muscle) [52]	↑Beta cells [58]	HSCs (↓IL-1α, ↓c-fos, ↓NF-κB) [40] MSCs (↑IL-6) [53]	Hepatic cancer stem cells [54] Breast cancer [55] Leukemia [56]	↑T cell receptor sensitivity [57] ↑Blood-brain barrier destruction [59]

↑: promotion/positive regulation, ↓: inhibition/negative regulation, Ad: adipocytes, Os: Osteoblasts, N: neurons, G: glial cells, B: B cells, Ly: lymphocytes, My: myeloid cells, Epi: epidermal cells, Oil: oil-gland cells, HF: hair follicle cells

## Conclusions

Some microRNAs have similar functions in different types of TSCs. Downregulation of these specific-microRNAs induces similar age-related dysfunctions of TSCs. These microRNAs may define the “young competence” by specifying the signal pathways with suppression of their regulon, including signal mediators and transcription factors. Further investigation of the roles of the other microRNAs in stem cell aging will help to elucidate the central molecular machinery of the aging and develop the next-generation therapeutic methods for geriatric diseases.

## Abbreviations

Gdf6: Growth differentiation factor 6
HSCs: Hematopoietic stem/progenitor cells
miR-17/106: miR-17, miR-106a, and 106b
MSCs: Mesenchymal stem/stromal cells
NSCs: Neural stem/progenitor cells
SASP: Senescence-associated secretory phenotype
TSCs: Tissue stem cells
